# Salter-Harris Type III and Type IV Combined Fracture of the Distal Femoral Epiphysis: A Case Report

**DOI:** 10.1155/2012/317848

**Published:** 2012-05-13

**Authors:** Ali Aydin, Murat Topal, Kutsi Tuncer, Eyüp Şenocak

**Affiliations:** Department of Orthopaedics and Traumatology, Atatürk University, Medical School Hospital, 25240 Erzurum, Turkey

## Abstract

Distal femoral physeal fractures are not common but have a high rate of complications. They generally follow one of the patterns described in the Salter-Harris classification. We present a case of combination of Salter-Harris type III and type IV injury. Our case was a 15-year-old boy who had a motor vehicle accident. There was swelling, ecchymosis, severe pain, and valgus deformity, because of medial proximal fracture fragment, on the left knee. We deemed that Salter-Harris type III and type IV combination fracture in our case has not been previously reported. We prepared this paper in consideration of its contribution to the literature.

## 1. Introduction

 Distal femoral epiphysis comes up in the ninth week of the fetal life and is the only epiphysis with a visible ossific nucleus at birth. Also, it is the biggest and the fastest growing epiphysis of the body, and it contributes to 40% of the lower extremity length [1]. The most common causes of injuries are traffic accidents and sport activities [2]. Distal femoral physeal fractures are not common but have a high rate of complications [3] and are responsible for 1–6% of all physeal injuries and less than 1% of fractures in children [4]. They generally follow one of the patterns described in the Salter-Harris classification [5]. Salter-Harris type II fractures are the most common fracture type of the distal femoral physis [3, 6]. Long-term complications like growth disturbance, with subsequent development of leg length discrepancy and/or angular deformities, are well reported to be seen in these certain types of injuries [3]. Both Salter-Harris classification and displacement of the fracture are significant predictors of the final outcome. The treatment method may influence the final outcome [3].

## 2. Case Presentation

**Figure 1:**
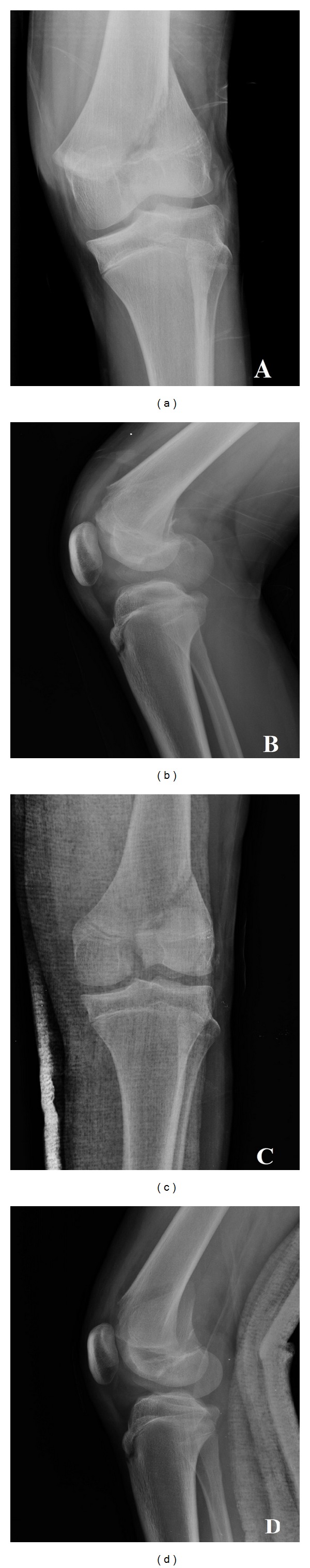
Anteroposterior and lateral radiograph of the left knee: Salter-Harris type III fracture of the medial physis and epiphysis, Salter-Harris type IV fracture of the lateral physis and epiphysis (a-b). Anteroposterior and lateral radiograph of the left knee after closed reduction. Salter-Harris type III fracture of the medial physis seems reduced, whereas Salter-Harris type IV fracture seems diplaced. (c-d).

**Figure 2:**
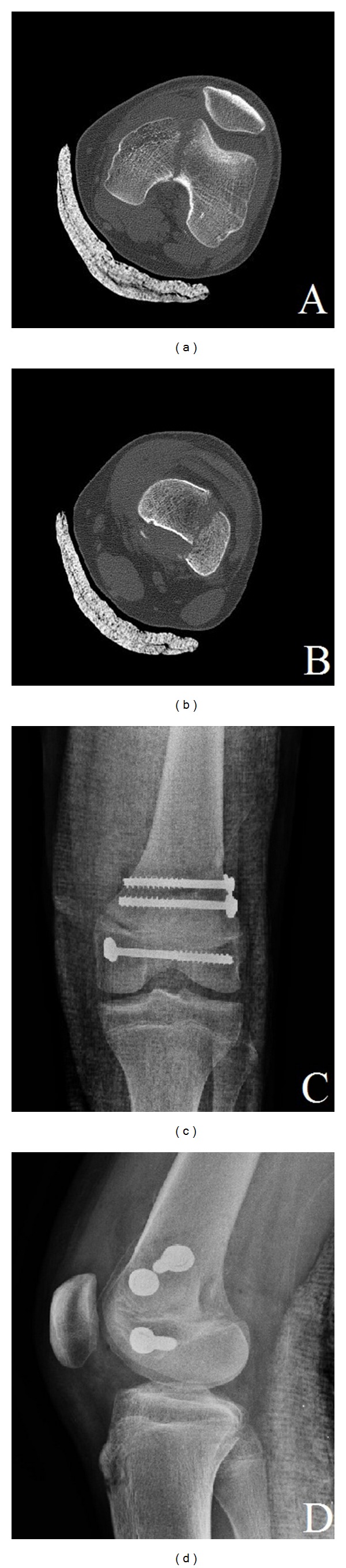
Computed tomography of physeal (a) and metaphyseal (b) fracture. Postoperative anteroposterior and lateral radiograph of the left knee (c-d).

Our case was a 15-year-old boy who had a motor vehicle accident. There was swelling, ecchymosis, severe pain, and valgus deformity, because of medial proximal fracture fragment, on the left knee. After evaluating the first radiograph (Figures 1(a) and 1(b)), closed reduction and long leg splint were done, and control X-ray was taken (Figures 1(c) and 1(d)). Correlation with computed tomography was performed in order to ascertain the fracture pattern (Figures 2(a) and 2(b)). The patient had no neurovascular problems on his first physical examination.

The fracture pattern was determined, and the patient underwent open reduction and internal fixation operation in emergency conditions. After a full anatomic reduction was achieved in our operation, the metaphyseal fragment was fixed to physis by two transverse cannulated screws. Fixation was also applied to the medial condyle with one cannulated screw (Figures 2(c) and 2(d)). Since the patient's growth continues, we ensured that no screw passes through the physis. The patient was followed up for three weeks with long leg splint in the postoperative period. At the end of the three weeks, the splint was removed, a knee exercise program was implemented, and the patient was asked to walk on tiptoes for three weeks. At the end of the six weeks, knee extension was full, flexion was 150°, and the patient started to walk with full weight. In his last control at the end of the one year, the knee regained full extension and 160° flexion.

## 3. Discussion

The distal femoral physis is the fastest growing growth plate in the human body at a rate of 1.0 cm per year, producing 70% of the longitudinal growth of the femur and 40% of the overall growth of the lower extremity [7, 8]. Physeal closure and cessation of growth typically occurs at an age between 14 and 16 years in girls and between 16 and 18 years in boys [1, 9]. Distal femoral epiphyseal fractures are uncommon but have a high incidence rate of complications [3] and are responsible for 1–6% of all physeal injuries and less than 1% of fractures in children [4]. Motor vehicle accidents and sports-related injuries are reported as the most common causes [2]. The Salter-Harris classification continues to be the most widely used classification system for physeal fractures [5]. It helps in understanding the mechanism of injury and in predicting the likelihood of complications. Salter-Harris type II fractures are the most common fracture type of the distal femoral physis [3, 6, 10]. Poor results correlated with severely displaced fractures, nonanatomic reduction, associated injuries, and open fractures. Complications included growth arrest, resulting in leg length discrepancy, permanent decreased range of motion, and angular deformity [2, 6]. Salter-Harris type I fractures had the lowest incidence of growth disturbance (36%), whereas Salter-Harris type IV fractures had the highest rate of growth disturbance at 64% [10]. In our case, it was a Salter-Harris type III and type IV combination, and there was a high complication risk due to the existence of displacement, and relatives of the patient were throughly informed about this.

The peroneal nerve is reported to be involved in about 3% of cases of distal femoral epiphyseal injuries [4]. The direction of displacement has been associated with the development of neurovascular injuries, where anterior displacement puts the popliteal artery at risk, and varus displacement places the peroneal nerve at risk [2]. In our case, no deficit existed in neurovascular structures.

Our case is a previously unpublished, Salter-Harris type III and type IV combination. We consider that high complication risks of distal femoral physeal fractures should be told to relatives of the patient. In order to decrease this high risk of complication, we consider that full anatomic reduction should be achieved by a sufficiently stabile fixation, and joint movement should be gained in early period.
